# Evaluating the prognostic value of miR-148/152 family in cancers: based on a systemic review of observational studies

**DOI:** 10.18632/oncotarget.20830

**Published:** 2017-09-11

**Authors:** Fujiao Duan, Weigang Liu, Xiaoli Fu, Yajing Feng, Liping Dai, Shuli Cui, Zhenxing Yang

**Affiliations:** ^1^ Medical Research Office, Affiliated Cancer Hospital of Zhengzhou University, Zhengzhou, Henan, China; ^2^ College of Public Health, Zhengzhou University, Zhengzhou, Henan, China; ^3^ Medical Record Statistics Office, Affiliated Hospital of Hebei University of Engineering, Handan, Hebei, China; ^4^ Department of Nosocomial Infection Management, The First Affiliated Hospital of Zhengzhou University, Zhengzhou, Henan, China; ^5^ College of Professional Study, Northeastern University, Boston, Massachusetts, USA; ^6^ Henan Key Laboratory of Tumor Epidemiology, Zhengzhou, Henan, China

**Keywords:** miR-148/152 family, prognosis, cancer, systems assessment

## Abstract

**Background:**

The prognostic significance of MicroRNA-148/152 (miR-148/152) family expression in various cancers has been investigated by many studies with inconsistent results. To address this issue, we performed a meta-analysis to clarify this relationship.

**Materials and Methods:**

Eligible studies were recruited by a systematic literature search and assessed the quality of included studies based on Quality In Prognosis Studies (QUIPS) and Newcastle-Ottawa Scale (NOS). Pooled hazard ratios (HRs) with 95% confidence intervals (CIs) for overall survival (OS) and disease free survival/progressive free survival/recurrence free survival (DFS/PFS/RFS) were calculated to estimate the effects of miR-148/152 family expression on prognosis.

**Results:**

A final total of 23 articles (26 studies) were considered in evidence synthesis. A significant association was observed between low miR-148a level and poor OS in patients (HR = 1.59, 95% CI: 1.14 – 2.20, *P* = 0.00), especially with digestive tract cancer (DTC) (HR = 1.29, 95% CI: 1.03–1.63, *P* = 0.03), and another significant association was observed between low miR-148b level and poor OS in patients (HR=2.09, 95% CI: 1.70–2.56, *P* = 0.00), especially with (hepatocellular carcinoma) HCC (HR = 1.97, 95% Cl: 1.52–2.56, *P* = 0.00) and non-small cell lung cancer (NSCLC) (HR = 2.29, 95% Cl: 1.64–3.18, *P* = 0.00). The significant correlation between miR-152 and DFS/RFS was found in our research (HR = 3.49, 95% Cl: 1.13–10.08, *P* = 0.03).

**Conclusions:**

Our findings suggest that low miR-148/152 family expression is significantly associated with poor prognosis and may be a feasible prognostic biomarker in some cancers, especially in HCC and NSCLC.

## INTRODUCTION

Cancer is a worldwidely major problem affecting public health [1]. In 2016, 1,685, 210 new cancer cases and 595,690 cancer deaths are projected to occur in the United States [2]. In China, cancer incidence and mortality have been increasing, making cancer the leading cause of death since 2010 [3]. Much of the rising burden of disease is attributable to the occurrence of cancers.

Many tumors express the miR-148/152 family differently in the process of tumorigenesis. MicroRNA (miRNA) is a class of evolutionarily conserved, single-stranded, non-coding RNA molecule (containing about 22 nucleotides) [4], it is estimated to regulate 30 % of all genes in animals by binding to specific sites in the 30 untranslated regions (30UTR), resulting in RNA silencing or post-transcriptional regulation of gene expression [5].

MiRNA-148 (MiR-148) and miR-152 are members of the miR-148/152 family, which consists of miR-148a, miR-148b and miR-152 [6]. The pre-miR-148/152 family have a stem-loop structure, which can be processed into the mature miR-148/152 family by a set of intracytoplasmic enzymes and intranuclear [7]. Mature miR-148/152 family is 21–22 nucleotides in length, with the same seed sequence of about 6–7 nucleotides, which is an important region for binding to target mRNAs [8]. Researches have found that mature miR-148/152 family can involve in different tumor biological processes through complementary binding between the seed sequence and the 30UTR of target mRNAs [9]. Therefore, miR-148/152 family might be critical for these processes.

Due to the diverse and crucial roles of miRNAs in tumors, clarifying the prognostic significance and exploring the complex function in various human cancer tissues about tumorigenesis and/or tumor suppression of the miR-148/152 family may provide constructive insights to efficacious cancers management.

In the present study, a systematic review with the data available from studies published in this field was carried out. We mainly focus on the expression level of miR-148a/b which can be used as prognostic classifiers to guide therapeutic decisions.

## MATERIALS AND METHODS

### Data sources and search strategy

### Ethics committee is not applicable in this study

The present study is conducted in accordance with Preferred Reporting Items for Systematic Review and Meta-analyses (PRISMA) guidelines [10] and the Meta-analysis of Observational Studies in Epidemiology group (MOOSE) issued by Stroup et al. [11].

We conducted a computerized literature search on multiple databases including PubMed, EMBASE and Web of Science through March 2017. The search items were combinations of “microRNA-148a” or “miR-148a”, “microRNA-148b” or “miR-148b”, “microRNA-152” or “miR-152” and “neoplasms” or “cancer”. We also searched the Google Scholar, Chinese National Knowledge Infrastructure (CNKI) and Wanfang database following the same keywords as assistance. We also manually searched original studies on this topic to further identify potentially relevant articles that may have been missed by the computerized search.

### Study selection and exclusion criteria

Eligible studies will be included in the present study if they were: (i) cohort studies assessing the prognostic significance of miR-148/152 family detected in patients with cancer; (ii) reported survival outcome or provided suffcient data to extrapolate the corresponding outcome measures (hazard ratios, HRs and 95% confidence intervals, 95% CIs); (iii) measured in cancer tissue or serum; and (iv) available in English or Chinese.

The exclusion criteria included: (i) reviews, non-human research, comments letters or laboratory studies; (ii) non- Chinese or English articles; (iii) redundant publications using the same population; and (iv) lacked key information regarding survival outcomes, such as HRs or 95%CIs or unable to calculate such parameters. If a study had overlapping data with other studies, we kept the study with larger sample size.

The retrieved articles were assessed for inclusion by FJD and YJF independently and discrepancies were resolved via discussion or consensus.

### Data extraction

Two independent reviewers (FJD and ZXY) identified eligibility studies using this search strategy to generate a list of potentially relevant articles, and to carry on data extraction and quality evaluation. Discrepancies were resolved by consensus.

The following characteristics and numbers were collected from each eligible study if they were available: first author, publication year, country of origin, histological classification, TNM stage, sample type and size, detection method, follow-up and value of cutoff, HRs of miR-148/152 family for overall survival (OS) and/or progressive free survival (PFS), recurrence free survival (RFS), disease free survival (DFS) and the corresponding 95% CIs, all these results were considered as independent data sets.

If data not reporting, the HR and 95% CI were extrapolated using the methods of Parmar [12] and Tierney [13].

### Quality assessment

Quality assessment criteria were utilized to evaluate methodological quality of included studies based on Newcastle-Ottawa Scale (NOS) [14]. The instrument rates observational studies on a nine-point scale, and the maximum score was nine, a high-quality study was defined as one with a score of ≥ 6. Discrepancies were resolved through consensus.

The specific Quality In Prognosis Studies (QUIPS) was evaluated according to the approach of Hayden et al. [15]. The estimated items with potential bias included study participation, study attrition, prognostic factor measurement, outcome measurement, study confounding, statistical analysis and reporting. The assessments were processed independently by two authors (XLF and KJW) and the final decision was achieved by consensus or consultation of a third party.

### Data synthesis and statistical analysis

We utilized RevMan 5.3.5 software (Version 5.3.5 for Windows, Cochrane Collaboration, Oxford, UK) and STATA software version 13.1MP (StataCorp, College Station, TX, USA) to perform this meta-analysis. HRs and corresponding 95% CIs were used to estimate the relationship strength between miR-148/152 family expression and patients’ prognosis. Cochran's Q test and Higgin's *I*^2^ statistic was utilized to measure between-study heterogeneity. If heterogeneity did exist (Pheterogeneity < 0.05 or *I*^2^ > 50%), random-effects model (DerSimonian and Laird method) [16] was used to calculate pooled HR, and meta-regression were further applied to investigate sources of heterogeneity [17]. If not, fixed-effects model (Mantel-Haenszel method) [18] was applied. The stratified assessments were conducted by ethnicity (Caucasian, Asian) and cancer subtypes, if one cancer type included no more than two individual studies, it was combined into the ‘other cancers’ group.

To assess the influence of selected studies on the pooled results, one-way sensitivity analyses were performed, and then by omitting each study in turn to assess the quality and consistency of the results.

Publication bias was evaluated using Begg's test (rank correlation test) [19] and Egger's test (weighted linear regression test) [20]. An asymmetric funnel plot would suggest the possibility of small studies not being published due to unfavorable results.

The significance of pooled HR was determined by the *Z*-test, *P* < 0.05 was considered statistically significant, all *P* values were two-sided.

## RESULTS

### Literature search and study characteristics

**Figure 1:**
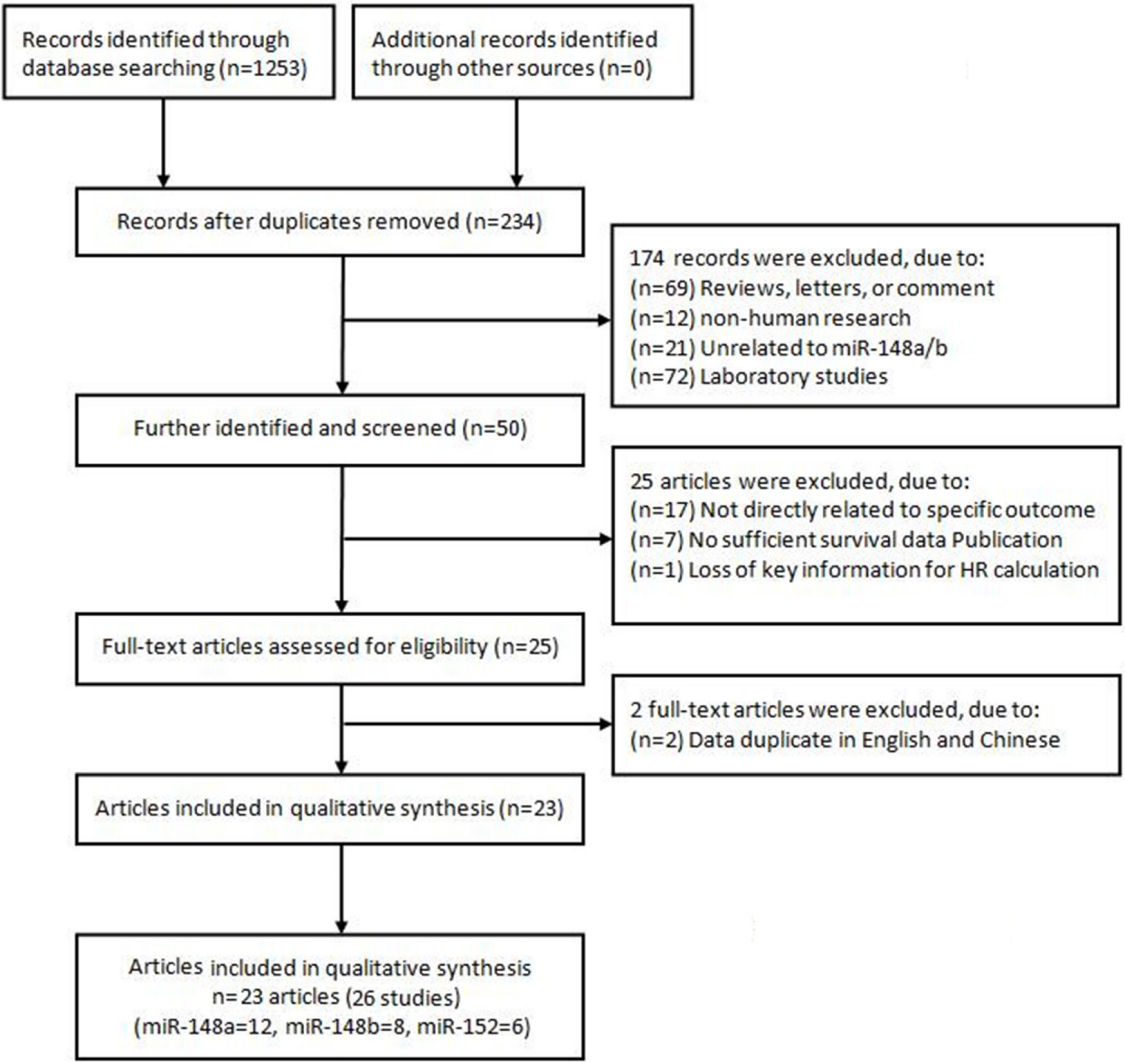
Flow chart of literature search and study selection

The search process and the final selection of relevant studies are shown in Figure 1 and a total of 1253 studies were identified by cautious searching and screening strategies. After excluding of duplicated studies, the remaining articles were 234. According to the exclusion criteria, 174 articles were further removed based on title or abstract screening. After further identification the individual study. According to the inclusion criteria, 25 articles [21–45] underwent full-text assessment, and two articles [21, 22]were excluded due to data duplication [26, 28]. Finally, a total of 23 articles [23–45] (26 studies), including 12 for miR-148a [23–34], 8 for miR-148b [26, 35–41] and 6 for miR-152 [26, 40, 42–45] respectively were included in evidence synthesis.

The baseline characteristics of eligible studies were summarized in Table 1. These eligible studies were published from 2010 to 2016 and included a total of 2641 patients from China, Korea, Norway, Spain, Denmark, America, Iran, France and Tohoku. The patients were classified Asian or Caucasian according to their ethnic background. The types of carcinomas in these studies included ovarian cancer, osteosarcoma, gastric cancer (GC), colorectal cancer, hepatocellular carcinoma (HCC), esophageal squamous cell carcinoma (ESCC), non-small cell lung cancer (NSCLC), endometrial serous adenocarcinoma (ESC) and pancreatic cancer. The detection method of miR-148/152 family were quantitative real-time polymerase chain reaction (qRT-PCR) in 26studies, and the remaining one study was Microarray. MiR-148/152 family expression levels were measured in tissue or Serum. The cutoff values of miR-148/152 family vary between the different studies, most with normal or median.

**Table 1:** Clinicopathological characteristics of eligible studies

Author	Year	Country	Ethnicity	Locus	Number		Histology	TNM Stage	Sample	Assay	Follow-up (Months)	Cut-off	Hazard ratios
					OS	DFS/PFS/RFS							
Gong [23]	2016	China	Asian	148a	102		Ovarian cancer	I-IV	Serum	qRT-PCR	60	Normal	HR/SC
Zhang [24]	2016	China	Asian	148a	92		Osteosarcoma	I-III	Frozen tissue	qRT-PCR	43	Median	SC
Qiu [25]	2016	China	Asian	148a	238		Gastric cancer	I-IV	Frozen tissue	qRT-PCR	84	Median	HR/SC
Wang F [26]	2016	China	Asian	148a,148b,152	76		HCC	I-IV	Serum	qRT-PCR	36	Median	HR/SC
Ma [27]	2016	China	Asian	148a	126		Bladder Cancer	I-III	Frozen tissue	qRT-PCR	120	Median	HR/SC
Ma [28]	2014	China	Asian	148a	89	PFS,89	Osteosarcoma	NA	Serum	qRT-PCR	97	Normal	HR/SC
Heo[29]	2014	Korea	Asian	148a	59	RFS,59	HCC	I-IV	Frozen tissue	qRT-PCR	76	Normal	SC
Kjersem [30]	2014	Norway	Caucasian	148a	150	PFS,150	Colorectal cancer	NA	Serum	qRT-PCR	60	Median	HR
Li [31]	2014	China	Asian	148a	75		ESCC	I-III	Frozen tissue	qRT-PCR	47	Median	SC
Takahashi [32]	2012	Spain	Caucasian	148a	201	DFS,200	Colorectal cancer	I-IV	Frozen tissue	qRT-PCR	144	Median	HR/SC
Schultz [33]	2012	Denmark	Caucasian	148a	256		Pancreatic Cancer	NA	Frozen tissue	qRT-PCR	196	Median	HR/SC
Huang [34]	2012	China	Asian	148a		RFS,40	Multiple myeloma	I-III	Frozen tissue	Microarray	52	Normal	SC
Wang RF [35]	2016	China	Asian	148b	65		NSCLC	I-IV	Frozen tissue	qRT-PCR	60	Median	HR/SC
Benson [36]	2015	America	Caucasian	148b		PFS,17	Ovarian Cancer	NA	Serum	qRT-PCR	25	Median	SC
Ziari [37]	2015	Iran	Caucasian	148b	101		HCC	I-IV	Frozen tissue	qRT-PCR	92	Normal	HR/SC
Ge [38]	2015	China	Asian	148b	151		NSCLC	I-IV	Frozen tissue	qRT-PCR	60	Normal	HR/SC
Zhang [39]	2015	China	Asian	148b	40		HCC	I-III	Frozen tissue	qRT-PCR	48	Normal	SC
Jiang [40]	2015	China	Asian	148b,152		RFS,252	Bladder cancer	NA	Serum	qRT-PCR	48	Normal	HR/SC
Zhang [41]	2014	China	Asian	148b	156		HCC	I-IV	Frozen tissue	qRT-PCR	60	Median	HR/SC
Wang Y [42]	2016	China	Asian	152	202		Colorectal cancer	I-IV	Frozen tissue	qRT-PCR	48	Median	HR/SC
Wang NG [43]	2015	China	Asian	152	80		Osteosarcoma	I-III	Frozen/ Tissue	qRT-PCR	60	Normal	HR/SC
Sanfiorenzo [44]	2013	France	Caucasian	152		DFS,52	NSCLC	I-III	Frozen tissue	qRT-PCR	66	Median	HR/SC
Hiroki [45]	2010	Tohoku	Asian	152	21	DFS,21	ESC	I-IV	Frozen tissue	qRT-PCR	72	Median	HR/SC

HCC, hepatocellular carcinoma; ESCC, esophageal squamous cell carcinoma; NSCLC, non-small cell lung cancer; ESC, endometrial serous adenocarcinoma; qRT-PCR, quantitative real-time PCR; OS, overall survival; PFS, progressive free survival; DFS, disease free survival; RFS, recurrence free survival; SC, survival curve.

### Qualitative assessment

**Figure 2:**
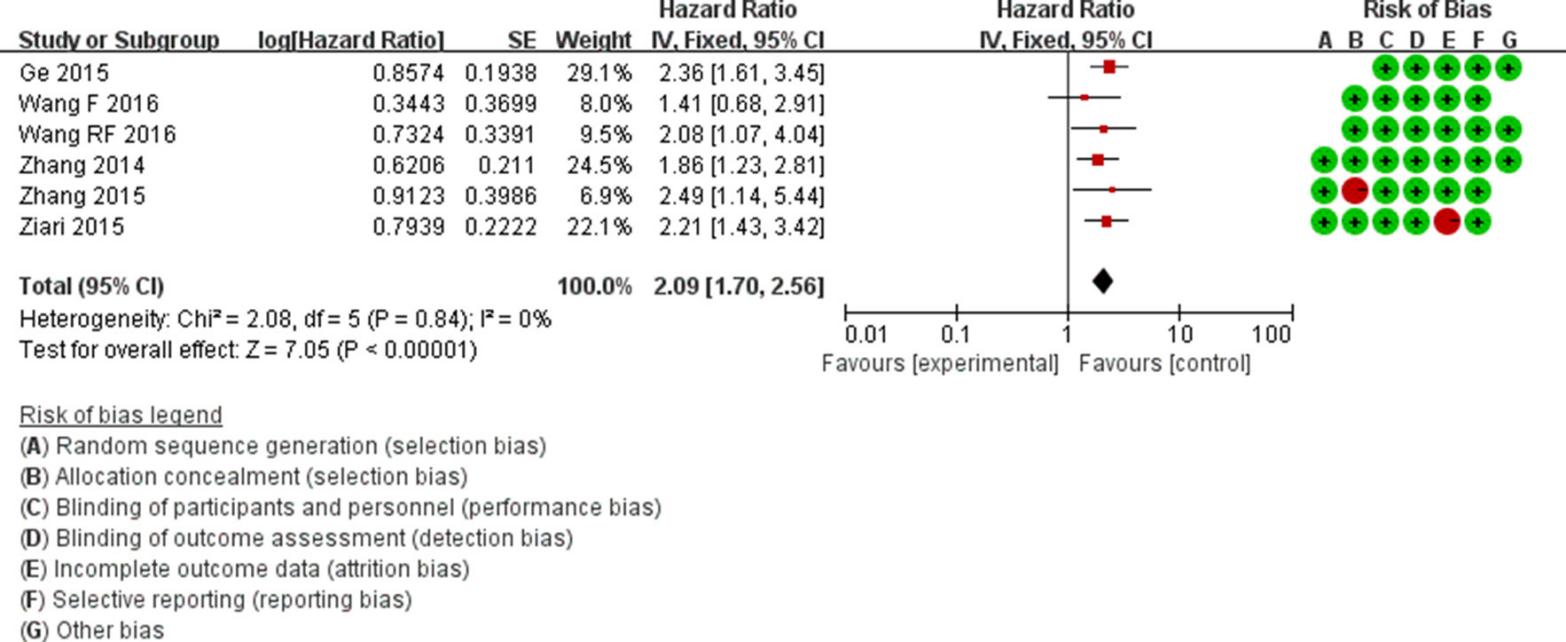
Forest plots of studies evaluating the HRs of high and low miR-148b expression with respect to OS

Based on the QUIPS Tool, the Table 2 summarizes the 6 bias domains (participation, attrition, prognostic factor measurement, confounding measurement and account, outcome measurement, and analysis and reporting) and the risk of bias legend in Figure 2. According to the NOS (Supplementary Table 1), seventy-eight percent (18/23) of these articles were high-quality (quality score ≥ 6).

**Table 2:** Quality assessment of included studies based on the Quality In Prognosis Studies (QUIPS)

Study	Quality evaluation of prognosis study						Total Score^a^	Level of Evidence^b^
	Study Participation	Study Attrition	Prognostic Factor Measurement	Outcome Measurement	Study Confounding	Statistical Analysis and Reporting		
Gong 2016 [23]	Yes	Partly	Yes	Yes	Partly	Yes	**6**	**2b**
Zhang 2016 [24]	Partly	Partly	Partly	Partly	Partly	Partly	**5**	**2b**
Qiu 2016 [25]	Partly	Partly	Yes	Yes	Partly	Yes	**6**	**2b**
Wang F 2016 [26]	Partly	Partly	Yes	Yes	Partly	Yes	**6**	**2b**
Ma 2016 [27]	Yes	Partly	Yes	Yes	Partly	Yes	**7**	**2b**
Ma 2014 [28]	Yes	Partly	Yes	Yes	Partly	Yes	**8**	**2b**
Heo 2014 [29]	Partly	Partly	Yes	Partly	Partly	Partly	**7**	**2b**
Kjersem 2014 [30]	Yes	Partly	Yes	Yes	Partly	Partly	**7**	**1b**
Li 2014 [31]	Yes	Yes	Yes	Yes	Partly	Yes	**8**	**2b**
Takahashi 2012 [32]	Yes	Yes	Yes	Yes	Partly	Yes	**8**	**1b**
Schultz 2012 [33]	Yes	Yes	Partly	Partly	Partly	Yes	**5**	**2b**
Huang 2012 [34]	Partly	Partly	Partly	Partly	Partly	Partly	**5**	**2b**
Wang RF 2016 [35]	Partly	Yes	Yes	Yes	Partly	Yes	**7**	**2b**
Benson 2015 [36]	Yes	Yes	Yes	Yes	Partly	Yes	**6**	**1b**
Ziari 2015 [37]	Yes	Partly	Partly	Yes	Partly	Yes	**7**	**2b**
Ge 2015 [38]	Partly	Partly	Yes	Yes	Partly	Yes	**6**	**2b**
Zhang 2015 [39]	Partly	Partly	Partly	Yes	Partly	Yes	**4**	**2b**
Jiang 2015 [40]	Yes	Partly	Yes	Yes	Partly	Yes	**5**	**2b**
Zhang 2014 [41]	Yes	Yes	Yes	Yes	Partly	Yes	**8**	**2b**
Wang Y 2016 [42]	Yes	Yes	Yes	Yes	Partly	Partly	**8**	**2b**
Wang NG 2015 [43]	Yes	Partly	Yes	Yes	Partly	Yes	**7**	**2b**
Sanfiorenzo 2013 [44]	Yes	Partly	Yes	Partly	Partly	Yes	**6**	**2b**
Hiroki 2010 [45]	Yes	Yes	Yes	Yes	Partly	Yes	**6**	**1b**

^a^Quality assessment of included studies based on the Newcastle-Ottawa Scale.

^b^The levels of evidence were estimated for all included studies with the Oxford Centre for Evidence Based Medicine criteria.

### Overall analyses

For the miR-148a, HRs for OS were provided by 11 studies, a significant association was observed between low miR-148a level and poor OS in patients (pooled HR = 1.59, 95% CI: 1.14–2.20, *P* = 0.00) Supplementary Figure 1. HRs for disease progression (DFS/PRS/RFS) were provided by 5 studies, no significantly correlation between miR-148a and DFS/PRS/RFS has been found (HR = 0.90, 95% Cl: 0.47–1.76, *P* = 0.77) (Table 3). Subgroup analysis was carried out by ethnicity. The expression of miR-148a was not significantly correlated with OS in Asain (HR = 1.61, 95% Cl: 0.87–2.95, *P* = 0.13) and Caucasian (HR = 1.13, 95% Cl: 0.84–1.51, *P* = 0.43). Similarly, miR-148a expression was not significantly correlated with DFS/PRS/RFS in Asain (HR = 0.71, 95% Cl: 0.15–3.27, *P* = 0.66) and Caucasian (HR = 1.15, 95% Cl: 0.50–2.67, *P* = 0.34) (Table 3). Furthermore, subgroup analysis was performed according to cancer subtypes, the results showed that a low expression level of miR-148a significantly predicted poor OS in digestive tract cancer (DTC) (HR = 1.29, 95%CI: 1.03–1.63, *P* = 0.03). However, subgroup analysis by cancer subtypes, there was no significant risk association was observed in the DFS/PRS/RFS pooled analysis (Table 3).

**Table 3:** Main results of pooled HRs in the meta-analysis

Comparisons	Heterogeneity test			Summary HR (95% CI)	Hypothesis test		Patients	Studies
	*Q*	*P*	*I^2^* (%)		*Z*	*P*		
***MircroRNA-148a***								
***Total***								
OS	115.47	0.00	91	1.59 (1.14,2.20)	2.77	0.00	1464	11
DFS/PRS/RFS	24.78	0.00	84	0.90 (0.47,1.76)	0.30	0.77	538	5
***Ethnicity***								
OS								
Asian	88.47	0.00	91	1.61 (0.87,2.95)	1.52	0.13	857	8
Caucasian	11.40	0.00	82	1.13 (0.84,1.51)	0.79	0.43	607	3
DFS/PRS/RFS								
Asian	13.15	0.00	85	0.71 (0.15,3.27)	0.45	0.66	188	3
Caucasian	10.27	0.00	90	1.15 (0.50,2.67)	0.34	0.74	350	2
***Cancer subtypes***								
OS								
DTC	20.64	0.00	76	1.29 (1.03,1.63)	2.18	0.03	799	6
Other cancers	83.57	0.00	95	1.50 (0.52,4.36)	0.75	0.45	665	5
DFS/PRS/RFS								
DTC	16.90	0.00	88	0.61 (0.17,2.22)	0.75	0.45	409	3
Other cancers	7.19	0.01	86	1.51 (0.34,6.71)	0.54	0.59	129	2
***MircroRNA-148b***								
***Total***								
OS	2.08	0.84	0	2.09 (1.70,2.56)	7.05	0.00	589	6
DFS/PRS/RFS	1.36	0.24	26	1.13 (0.62,2.04)	0.40	0.69	269	2
***Cancer subtypes***								
OS								
HCC	1.51	0.68	0	1.97 (1.52,2.56)	5.10	0.00	373	4
NSCLC	0.10	0.75	0	2.29 (1.64,3.18)	4.91	0.00	216	2
***MircroRNA-152***								
***OS***	13.21	0.00	77	1.04 (0.26,4.17)	0.06	0.95	379	4
DFS/RFS	4.74	0.09	58	3.49 (1.13,10.83)	2.17	0.03	325	3

DTC, digestive tract cancer, including colorectal cancer, esophageal squamous cell carcinoma, pancreatic pancer and hepatocellular carcinoma.

For the miR-148b, HRs for OS were provided by 6 studies, a significant association was observed between low miR-148b level and poor OS in patients (pooled HR = 2.09, 95%CI: 1.70–2.56, *P* = 0.00) (Figure 2). HRs for disease progression (DFS/PRS/RFS) were provided by 2 studies, which indicated no significantly correlation between miR-148b expression and DFS/PRS/RFS (HR = 1.13, 95%Cl: 0.62–2.04, *P* = 0.69) (Table 3). Subgroup analysis was performed based on cancer subtypes, miR-148b expression was significantly correlated with OS in patients with HCC (HR = 1.97, 95%Cl: 1.52–2.56, *P* = 0.00) and NSCLC (HR = 2.29, 95%Cl: 1.64–3.18, *P* = 0.00) (Table 3).

For the miR-152, HRs for OS were provided by 4 studies, miR-152 expression was not significantly correlated with OS in cancer (HR = 1.04, 95%Cl: 0.26–4.17, *P* = 0.95). However, it was significantly correlated with DFS/RFS in cancer (HR = 3.49, 95%Cl: 1.13–10.86, *P* = 0.03). Due to the limited availability of eligible studies, stratified study hasn't been conducted (included studies populations were all Asians except one Caucasian) (Table 3).

### Meta-regression analysis

When evaluating the association between the miR-148/152 family expression and the risk of cancer, we found that there were significant heterogeneity among studies of miR-148a and miR-152, but we only evaluated the source of heterogeneity of miR-148a due to limited published data of miR-152. Thus, we conducted a meta-regression analysis to investigate potential source of heterogeneity by publication year, cancer types, ethnicity, languages, assays, sample sizes (100 as the boundary), quality (Based on NOS score). Meta-regression analysis indicated that the systemic outcomes were not affected by above characteristics (Table 4).

**Table 4:** Publication bias of miR-148a and mir-148b for Begg's test and Egger's test

Comparisons	Begg's test		Egger's test		
	*z*	*p*	*t*	*p*	95% CI
***MircroRNA-148a***					
OS	0.16	0.876	0.91	0.386	−1.970–4.627
DFS/PRS/RFS	0.24	0.806	0.25	0.820	0.591–6.947
***MircroRNA-148b***					
OS	0.38	0.707	−0.64	0.556	−3.514–2.193
DFS/PRS/RFS*					
***MircroRNA-152***					
OS	0.75	0.452	0.87	0.443	−3.328–6.367
DFS /RFS*					

* Insufficient observations.

### Sensitivity analyses and publication bias

**Figure 3:**
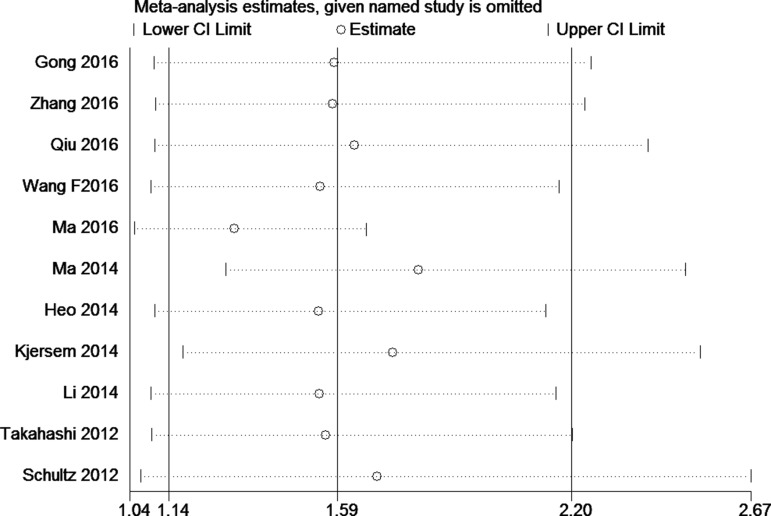
Sensitivity analysis for OS of miR-148a

Sensitivity analyses were carried out to assess the influence of each individual study by omitting individual data set, the results didn't alter materially, which indicated that pooled HRs were quite stable (Figure 3).

**Figure 4:**
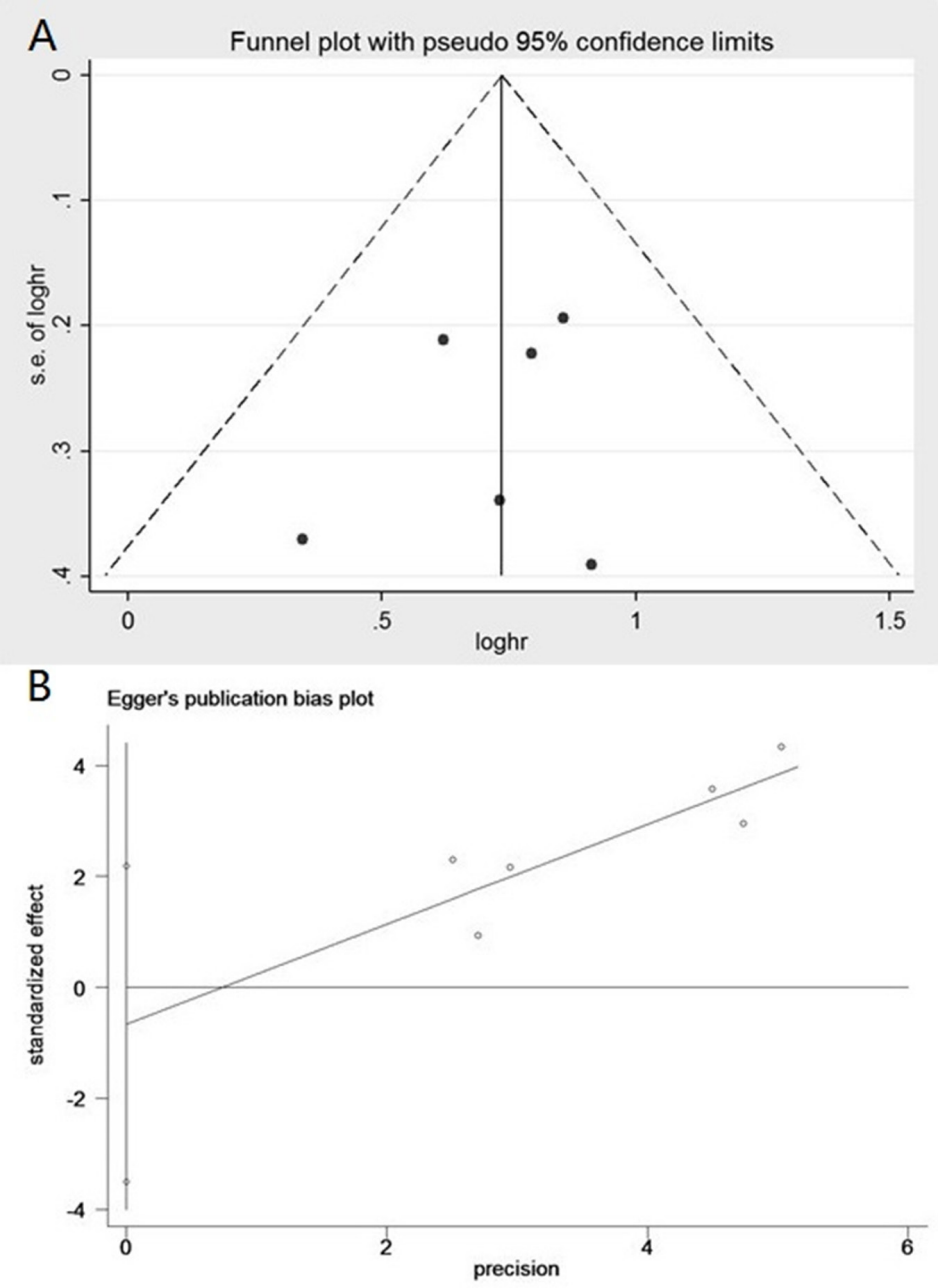
(**A**) Begg's funnel plot of publication bias on the relationship between miR-148b expression and OS. (**B**) Egger's funnel plot of publication bias. on the relationship between miR-148b expression and OS.

Begg's funnel plot and Egger's test were performed to assess the publication bias. The shape of the funnel plots did not reveal any visual evidence of the asymmetry, indicating that our results were statistically robust (Table 5, Figure 4A and 4B).

**Table 5:** The results of heterogeneity test

Comparisons	Coef.	Sth. Err.	*t*	*P*	95% CI
***MircroRNA-148a***					
Publication year	0.896	1.261	0.71	0.516	−2.603–4.396
Cancer type	0.0415	0.188	0.22	0.836	−0.481–0.565
Language	1.021	1.422	0.72	0.512	−2.925–4.396
Assay	−2.957	1.863	−1.59	0.188	−8.1299–2.214
Sample size	−1.057	1.222	−0.87	0.436	−4.449–2.335
Quality	0.044	0.803	0.06	0.958	−2.185–2.274
MircroRNA-148b*	-	-	-	-	-
MircroRNA-152*	-	-	-	-	-

* MircroRNA-148b was dropped because of insufficient observations.

## DISCUSSION

MiR-148/152 family members have aberrant expression in normal tissue, especially in stem cells [7, 46]. MiR-148a expression in hematopoietic stem cells (HSCs) was investigated and found that miR-148a was decreased in HSCs [47]. Qureshi et al. reported that the miR-148b was upregulated in osteogenesis of early osteogenic differentiation of human mesenchymal stem cells [48]. Manaster et al. reported that in placental tissue, miR-152 was expressed at relatively low levels compared with other healthy tissues. In addition, miR-152 as a member of miRNAs was found with aberrant expression levels in different malignant tumors [43]. Therefore, miR-148/152 family may serve as potential biomarkers to indicate different tumor courses and outcomes.

MiR-148/152 family members are decreased in different types of cancer, indicating that they have the potential to act as tumor-suppressors. Li et al. found miR-148b was downregulated in liver cancer stem cells (LCSCs) [49]. Besides, Huang et al. demonstrated that miR-152 was underexpressed in HBV-related HCC tissues compared with the adjacent noncancerous hepatic tissues. Chen et al. found that low expression of miR-148a and miR-152 correlated with increased tumor size and advanced pT stage [6]. Furthermore, they suggested that miR-148a and miR-152 were downregulated in cancer cell lines and cancer tissue [6].

To the best of our knowledge, our study is the first to critically examine available literature and identify the prognostic role of miR-148/152 family in various cancers, which was evaluated by the pooled HRs from 26 published studies. In the present study, we initially performed a systematic review and meta-analysis to comprehensively and systematically evaluate the prognostic value of the miR-148/152 family expression in cancer patients. We found that lower levels of miR-148a and 148b were signifcantly associated with shorter OS, particularly in patients with HCC and NSCLC for miR-148b. Similarly, miR-152 was significantly correlated with DFS/RFS in cancer.

Published literature has confirmed that negative correlations between miR-148/152 family expression and tumor phenotypes of NSCLC, which may be better explanations for how all three miRNAs (miR-148/152 family) can function as cancer suppressors in NSCLC [50]. The functional assays demonstrated that miR-148a inhibits epithelial-to-mesenchymal transition (EMT) in NSCLC cells by a metastasis promoter of directly targeting coiled-coil containing protein kinase 1 (ROCK1) [51]. Meanwhile, miR-148b suppresses cell migration and proliferation in NSCLC cell lines by targeting carcino-embryonic antigen (CEA) [52]. In blood, circulating miRNAs are stabilized by interaction with microvesicles and RNA-binding proteins so as to resist the endogenous RNase activity, and exist as cell-free forms [53]. The miR-148b suppresses proliferation and invasion of HCC cells by direct targeting neuropilin-1, the molecules could be detected not only in in body fluids (serum, plasma, cerebral spinal fluid and urine) but also tumor tissues, it expression was decreased in HCC [54, 55]. Evidence has been increasing that miR-152 may act as a tumor suppressor gene by regulating corresponding target genes, which are associated with migration, cell proliferation and invasion in human cancer [8].

MiR-148/152 family is also regulated by other pathways. Zheng et al. reported a significant inverse association between miR-148a level and lymph node metastasis in GC, and implied that the invasion and migration of GC cells was suppressed via targeting Rho-associated, ROCK1 by miR-148a [56]. Moreover, Song et al. noted that miR-148b may act as a tumor suppressor in colorectal cancer and GC [57], and indicated that the suppression of tumor growth might be fulfilled by targeting cholecystokinin-2 receptor (CCK2R) [58].

Although meta-analysis is robust, several limitations should be addressed as follows. Firstly, although we find no evidence of publication bias, most included papers were English, which may generate publication bias. Secondly, due to not all the included studies provide multivariate adjusted HRs, in this case, some data was extracted from survival curves. These calculated HRs with the 95%CIs might be brought several tiny errors. Thirdly, the definition in miR-148/152 family cut-off is ambiguous. Although most of them defined median as the cut-off of elevated miR-148/152 family expression, the actual values may be various between the different study populations. Therefore, the present study could not establish the exact cut-off value. Fourthly, due to the limited availability of eligible studies, stratified study hasn't been conducted for miR-152. Finally, the influence of adjuvant therapies on the prognosis of cancers was not evaluated in this study due to few included studies provided such data. More large-scale and well-designed studies are required to update the findings of this meta-analysis. In spite of these limitations, our work is the first meta-analysis to assess the prognostic significance of miR-148/152 family expression of patients with cancer.

In conclusion, our findings demonstrate that low miR-148a/b family expression is significantly associated with poor prognosis and may be a suitable prognostic biomarker in some cancer types, especially in HCC and NSCLC. More multicenter and well-designed studies with larger sample sizes should be conducted to confirm and update these findings.

## SUPPLEMENTARY MATERIALS FIGURES AND TABLES



## Abbreviations

miRNAs: microRNAs
miR-148a: microRNA-148a
miR-148b: microRNA-148b
miR-152: microRNA-152
HRs: Hazard ratios
CIs: confidence intervals
DFS: disease free survival
PFS: progressive free survival
RFS: recurrence free survival.

## References

[1] Siegel RL, Miller KD, Jemal A (2017). Cancer statistics, 2017. CA Cancer J Clin.

[2] Siegel RL, Miller KD, Jemal A (2016). Cancer statistics, 2016. CA Cancer J Clin.

[3] Chen W, Zheng R, Baade PD, Zhang S, Zeng H, Bray F, Jemal A, Yu XQ, He J (2016). Cancer statistics in China, 2015. CA Cancer J Clin.

[4] Sun K, Lai EC (2013). Adult-specific functions of animal microRNAs. Nature reviews genetics.

[5] Ambros V (2004). The functions of animal microRNAs. Nature.

[6] Chen Y, Song Y, Wang Z, Yue Z, Xu H, Xing C, Liu Z (2010). Altered expression of MiR-148a and MiR-152 in gastrointestinal cancers and its clinical significance. J Gastrointest Surg.

[7] Chen Y, Song YX, Wang ZN (2013). The MicroRNA-148/152 Family: Multi-faceted Players. Molecular Cancer.

[8] Liu X, Li J, Qin F, Dai S (2016). miR-152 as a tumor suppressor microRNA: Target recognition and regulation in cancer. Oncology Letters.

[9] Zhang Z, Zheng W, Hai J (2014). MicroRNA-148b expression is decreased in hepatocellular carcinoma and associated with prognosis. Medical Oncology.

[10] Moher D, Liberati A, Tetzlaff J, Altman DG (2009). Preferred reporting items for systematic reviews and meta-analyses: the PRISMA statement. Annals of internal medicine.

[11] Stroup DF, Berlin JA, Morton SC, Olkin I, Williamson GD, Rennie D, Moher D, Becker BJ, Sipe TA, Thacker SB (2000). Meta-analysis of observational studies in epidemiology: a proposal for reporting. Meta-analysis Of Observational Studies in Epidemiology (MOOSE) group. Jama.

[12] Parmar MK, Torri V, Stewart L (1998). Extracting summary statistics to perform meta-analyses of the published literature for survival endpoints. Stat Med.

[13] Tierney JF, Stewart LA, Ghersi D, Burdett S, Sydes MR (2007). Practical methods for incorporating summary time-to-event data into meta-analysis. Trials.

[14] Wells G, Shea B, O’connell D, Peterson J, Welch V, Losos M, Tugwell P (2014). The Newcastle-Ottawa Scale (NOS) for assessing the quality of nonrandomised studies in meta-analyses. http://www.ohri.ca/programs/clinical_epidemiology/oxford.asp.

[15] Hayden JA, Windt DAVD, Cartwright JL, Côté P, Bombardier C (2013). Assessing Bias in Studies of Prognostic Factors. Annals of internal medicine.

[16] DerSimonian R, Laird N (1986). Meta-analysis in clinical trials. Controlled clinical trials.

[17] Thompson SG, Higgins JP (2002). How should meta-regression analyses be undertaken and interpreted?. Stat Med.

[18] Mantel N, Haenszel W (1959). Statistical aspects of the analysis of data from retrospective studies of disease. Journal of the National Cancer Institute.

[19] Begg CB, Mazumdar M (1994). Operating characteristics of a rank correlation test for publication bias. Biometrics.

[20] Egger M, Davey Smith G, Schneider M, Minder C (1997). Bias in meta-analysis detected by a simple, graphical test. BMJ.

[21] Wang F, Sun H, Ying H, He B, Pan Y, Wang S (2016). Clinical value of serum miR-148a as a potential biomarker for hepatocellular carcinoma. Acta Universitatis Medicinalis Nanjing (Natural Science).

[22] Ma WL The research of the expression of miR-148a in patients with ostesarcoma and its function mechanism.

[23] Gong L, Wang C, Gao Y, Wang J (2016). Decreased expression of microRNA-148a predicts poor prognosis in ovarian cancer and associates with tumor growth and metastasis. Biomedicine & pharmacotherapy.

[24] Zhang H, Wang Y, Xu T, Li C, Wu J, He Q, Wang G, Ding C, Liu K, Tang H (2016). Increased expression of microRNA-148a in osteosarcoma promotes cancer cell growth by targeting PTEN. Oncology Letters.

[25] Qiu X, Zhu H, Liu S, Tao G, Jin J, Chu H, Wang M, Tong N, Gong W, Zhao Q (2017). Expression and prognostic value of microRNA-26a and microRNA-148a in gastric cancer. Journal of gastroenterology and hepatology.

[26] Wang F, Ying H, He B, Pan Y, Sun H, Wang S (2016). Circulating miR-148/152 family as potential biomarkers in hepatocellular carcinoma. Tumor Biology.

[27] Ma L, Xu Z, Xu C, Jiang X (2016). MicroRNA-148a represents an independent prognostic marker in bladder cancer. Tumor Biology.

[28] Ma WL, Zhang XH, Chai J, Chen P, Ren P, Gong MZ (2014). Circulating miR-148a is a significant diagnostic and prognostic biomarker for patients with osteosarcoma. Tumor Biology.

[29] Heo MJ, Kim YM, Koo JH, Yang YM, An J, Lee SK, Lee SJ, Kim KM, Park JW, Kim SG (2014). microRNA-148a dysregulation discriminates poor prognosis of hepatocellular carcinoma in association with USP4 overexpression. Oncotarget.

[30] Kjersem JB, Ikdahl T, Lingjaerde OC, Guren T, Tveit KM, Kure EH (2014). Plasma microRNAs predicting clinical outcome in metastatic colorectal cancer patients receiving first-line oxaliplatin-based treatment. Molecular Oncology.

[31] Li SH, Tian H, Yue WM, Li L, Gao C, Si LB, Hu WS, Yuan L, Lu M (2014). Clinical significance of mir-148a expression in patients with esophageal squamous cell cancer. Chin J Thorac Surg (Electronic Editon).

[32] Takahashi M, Cuatrecasas M, Balaguer F, Hur K, Toiyama Y, Castells A, Boland CR, Goel A (2012). The Clinical Significance of MiR-148a as a Predictive Biomarker in Patients with Advanced Colorectal Cancer. PloS one.

[33] Schultz NA, Andersen KK, Roslind A, Willenbrock H, Wøjdemann M, Johansen JS (2012). Prognostic microRNAs in cancer tissue from patients operated for pancreatic cancer--five microRNAs in a prognostic index. World Journal of Surgery.

[34] Huang JJ, Yu J, Li JY, Liu YT, Zhong RQ (2012). Circulating microRNA expression is associated with genetic subtype and survival of multiple myeloma. Medical Oncology.

[35] Wang R, Fan Y, Qiang Z, Song T, Tan G, Chu W, Zhang Y, Lv B, Zhao X, Liu J (2016). MicroRNA-148b is a potential prognostic biomarker and predictor of response to radiotherapy in non-small-cell lung cancer. Journal of Physiology and Biochemistry.

[36] Benson EA, Skaar TC, Liu Y, Nephew KP, Matei D (2015). Carboplatin with Decitabine Therapy, in Recurrent Platinum Resistant Ovarian Cancer, Alters Circulating miRNAs Concentrations: A Pilot Study. PloS one.

[37] Ziari K, Zarea M, Gity M, Fayyaz AF, Yahaghi E, Darian EK, Hashemian AM (2016). RETRACTED ARTICLE: Downregulation of miR-148b as biomarker for early detection of hepatocellular carcinoma and may serve as a prognostic marker. Tumor Biology.

[38] Ge H, Li B, Hu WX, Li RJ, Jin H, Gao MM, Ding CM (2015). MicroRNA-148b is down-regulated in non-small cell lung cancer and associated with poor survival. Int J Clin Exp Pathol.

[39] Zhang JG, Shi Y, Hong DF, Song M, Huang D, Wang CY, Zhao G (2015). MiR-148b suppresses cell proliferation and invasion in hepatocellular carcinoma by targeting WNT1/β-catenin pathway. Scientific reports.

[40] Jiang X, Du L, Wang L, Li J, Liu Y, Zheng G, Qu A, Zhang X, Pan H, Yang Y (2015). Serum microRNA expression signatures identified from genome-wide microRNA profiling serve as novel noninvasive biomarkers for diagnosis and recurrence of bladder cancer. International Journal of Cancer.

[41] Zhang Z, Zheng W, Hai J (2014). MicroRNA-148b expression is decreased in hepatocellular carcinoma and associated with prognosis. Medical Oncology.

[42] Wang Y, Yuan W, Ma X, Ma J (2016). Expression of microRNA-152 in colorectal cancer and its relationship with prognosis. Zhonghua zhong liu za zhi.

[43] Wang NG, Wang DC, Tan BY, Feng W, Yuan ZN (2015). Down-regulation of microRNA152 is associated with the diagnosis and prognosis of patients with osteosarcoma. International Journal of Clinical & Experimental Pathology.

[44] Sanfiorenzo C, Ilie MI, Belaid A, Barlési F, Mouroux J, Marquette CH, Brest P, Hofman P (2013). Two Panels of Plasma MicroRNAs as Non-Invasive Biomarkers for Prediction of Recurrence in Resectable NSCLC. PloS one.

[45] Hiroki E, Akahira J, Suzuki F, Nagase S, Ito K, Suzuki T, Sasano H, Yaegashi N (2010). Changes in microRNA expression levels correlate with clinicopathological features and prognoses in endometrial serous adenocarcinomas. Cancer science.

[46] Li L, Chen YY, Li SQ, Huang C, Qin YZ (2015). Expression of miR-148/152 Family as Potential Biomarkers in Non-Small-Cell Lung Cancer. Medical Science Monitor International Medical Journal of Experimental & Clinical Research.

[47] Báez A, Martín-Antonio B, Piruat JI, Barbado MV, Prats C, Álvarez-Laderas I, Carmona M, Pérez-Simón JA, Urbano-Ispizua Á (2014). Gene and miRNA Expression Profiles of Hematopoietic Progenitor Cells Vary Depending on Their Origin. Biol Blood Marrow Transplant.

[48] Qureshi AT, Monroe WT, Dasa V, Gimble JM, Hayes DJ (2013). miR-148b-nanoparticle conjugates for light mediated osteogenesis of human adipose stromal/stem cells. Biomaterials.

[49] Li R, Qian N, Tao K, You N, Wang X, Dou K (2010). MicroRNAs involved in neoplastic transformation of liver cancer stem cells. Journal of Experimental & Clinical Cancer Research.

[50] Li L, Chen Y, Li S, Huang C, Qin Y (2015). Expression of miR-148/152 Family as Potential Biomarkers in Non-Small-Cell Lung Cancer. Med Sci Monit.

[51] Li J, Song Y, Wang Y, Luo J, Yu W (2013). MicroRNA-148a suppresses epithelial-to-mesenchymal transition by targeting ROCK1 in non-small cell lung cancer cells. Molecular & Cellular Biochemistry.

[52] Liu GL, Liu X, Lv XB, Wang XP, Fang XS, Sang Y (2014). miR-148b functions as a tumor suppressor in non-small cell lung cancer by targeting carcinoembryonic antigen (CEA). International Journal of Clinical & Experimental Medicine.

[53] Jiang L, Cheng Q, Zhang BH, Zhang MZ (2015). Circulating microRNAs as biomarkers in hepatocellular carcinoma screening: a validation set from China. Medicine.

[54] Qi J, Wang J, Katayama H, Sen S, Liu SM (2013). Circulating microRNAs (cmiRNAs) as novel potential biomarkers for hepatocellular carcinoma. Neoplasma.

[55] Wang WT, Chen YQ (2014). Circulating miRNAs in cancer: from detection to therapy. Journal of Hematology & Oncology.

[56] Zheng B, Liang L, Wang C, Huang S, Cao X, Zha R, Liu L, Jia D, Tian Q, Wu J (2011). MicroRNA-148a suppresses tumor cell invasion and metastasis by downregulating ROCK1 in gastric cancer. Clin Cancer Res.

[57] Song YX, Yue ZY, Wang ZN, Xu YY, Yang L, Xu HM, Xue Z, Li J, Xing CZ, Yong Z (2011). MicroRNA-148b is frequently down-regulated in gastric cancer and acts as a tumor suppressor by inhibiting cell proliferation. Molecular Cancer.

[58] Song Y, Xu Y, Wang Z, Chen Y, Yue Z, Gao P, Xing C, Xu H (2012). MicroRNA-148b suppresses cell growth by targeting cholecystokinin-2 receptor in colorectal cancer. International journal of cancer.

