# Supplementary material for: Evaluating the prognostic value of miR-148/152 family in cancers: based on a systemic review of observational studies
Source: Oncotarget. 2017 Sep 11;8(44):77999–8010. doi: 10.18632/oncotarget.20830 (PMC5652831; doi:10.18632/oncotarget.20830)
Supplement: Supplementary file 1 [file oncotarget-08-77999-s001.pdf]

Evaluating the prognostic value of miR-148/152 family in cancers: based on a systemic review of observational studies

SUPPLEMENTARY MATERIALS

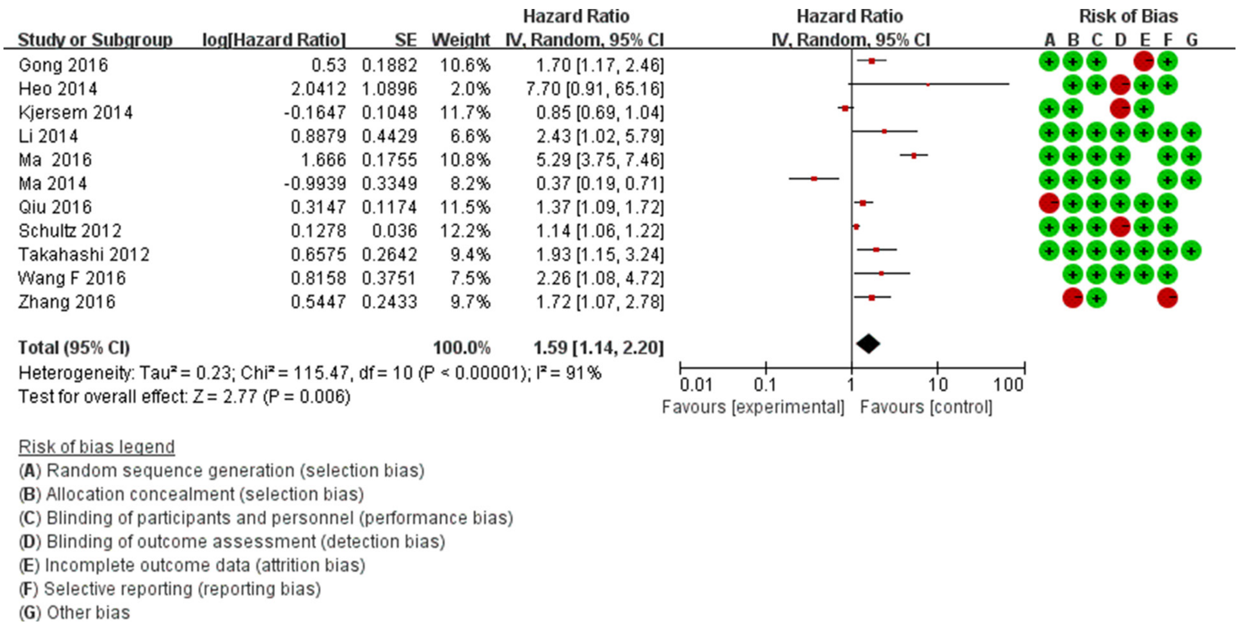

Supplementary Figure1: Forest plots of studies evaluating the HRs of high and low miR-148a expression with respect to OS.

Supplementary Table 1: Quality assessment of included studies based on the Newcastle–Ottawa Scale for assessing the quality of cohort studies

Study	Selection (score)				Comparability (score)	Exposure (score)			
	Representativenes Of the exposed cohort	Selection of the non-exposed cohort	Ascertainment of exposure	Outcome of interest was not present at start of study	Based on the design or analysis [a]	Assessment of outcome	Follow-up long enough for outcomes to occur	Adequacy of follow-up of cohorts	Total Score[b]
Gong 2016 [23]	1	0	0	1	2	0	1	1	**6**
Zhang 2016 [24]	1	0	0	1	0	1	1	1	**5**
Qiu 2016 [25]	1	0	0	1	2	0	1	1	**6**
Wang F 2016 [26]	1	0	1	1	2	0	0	1	**6**
Ma 2016 [27]	1	0	0	1	1	1	1	1	**6**
Ma 2014 [28]	1	0	1	1	1	1	1	1	**7**
Heo 2014 [29]	1	0	0	1	2	1	1	1	**7**
Kjersem 2014 [30]	1	0	0	1	2	1	1	1	**7**
Li 2014 [31]	1	0	1	1	2	1	1	1	**8**
Takahashi 2012 [32]	1	1	0	1	2	1	1	1	**8**
Schultz 2012 [33]	1	0	0	1	0	1	1	1	**5**
Huang 2012 [34]	1	0	0	1	0	1	1	1	**5**
Wang RF2016 [35]	1	0	0	1	2	1	1	1	**7**
Benson 2015 [36]	1	1	1	1	0	1	0	1	**6**
Ziari 2015 [37]	1	0	0	1	2	1	1	1	**7**
Ge 2015 [38]	1	0	1	1	1	0	1	1	**6**
Zhang 2015 [39]	1	0	0	1	0	0	1	1	**4**
Jiang 2015 [40]	1	0	0	1	0	1	1	1	**5**
Zhang 2014 [41]	1	1	0	1	2	1	1	1	**8**
Wang Y 2016 [42]	1	0	1	1	2	1	1	1	**8**
Wang NG 2015 [43]	1	0	0	1	2	1	1	1	**7**
Sanfiorenzo 2013 [44]	1	0	1	1	0	1	1	1	**6**
Hiroki 2010 [45]	1	1	0	1	0	1	1	1	**6**

[a]When there was no statistical significance in the response rate between case and control groups by using a chi-squared test ($P > 0.05$), one point was awarded.
[b]Total score was calculated by adding up the points awarded in each item.
